# Induction of a high-titered antibody response using HIV gag-EV71 VP1-based virus-like particles with the capacity to protect newborn mice challenged with a lethal dose of enterovirus 71

**DOI:** 10.1007/s00705-018-3797-7

**Published:** 2018-03-27

**Authors:** Xi Wang, Ke Dong, Min Long, Fang Lin, Zhaowei Gao, Lin Wang, Zhe Zhang, Xi Chen, Ying Dai, Huiping Wang, Huizhong Zhang

**Affiliations:** Department of Medical Laboratory and Research Center, Tangdu Hospital, Fourth Military Medical University, Xinsi Road No 569, 710038 Xi’an, Shaanxi China

## Abstract

Enterovirus 71 (EV71) is the most frequently detected causative agent in hand, foot, and mouth disease (HFMD) and is a serious threat to public health in the Asia-Pacific region. Many EV71 vaccines are under development worldwide, and although both inactivated virus vaccines and virus-like particles (VLPs) are considered to be effective, the main focus has been on inactivated EV71vaccines. There have been very few studies on EV71 VLPs. In this study, using a strategy based on HIV gag VLPs, we constructed a gag-VP1 fusion gene to generate a recombinant baculovirus expressing the EV71 structural protein VP1 together with gag, which was then used to infect TN5 cells to form VLPs. The VLPs were characterized using transmission electron microscopy, electrophoresis and staining with Coomassie blue, and Western blotting. Mice immunized with gag-VP1 VLPs showed strong humoral and cellular immune responses. Finally, immunization of female mice with gag-VP1 VLPs provided effective protection of their newborn offspring against challenge with a lethal dose EV71. These results demonstrate a successful approach for producing EV71 VP1 VLPs based on the ability of HIV gag to self-assemble, thus providing a good foundation for producing high-titered anti-EV71 antibody by immunization with VLP-based gag EV71 VP1 protein.

## Introduction

Human enterovirus 71 (EV71) is a non-enveloped RNA virus belonging to the family *Picornaviridae* that was first reported and then isolated in the year 1969 [1, 2]. It has been identified as one of the major causative agents of hand, foot, and mouth disease (HFMD), which mostly affects children under the age of six [2, 3]. Although most EV71 infections resolve spontaneously, they can occasionally cause severe neurological complications, which may lead to high morbidity rates [4, 5]. Several HFMD epidemics have occurred worldwide since its initial identification, particularly in the Asia-Pacific region [6, 7].

During the last 10 years, large-scale outbreaks of HFMD have been reported with increasing prevalence in China [8, 9]. The shortage of prophylactic and therapeutic measures against HFMD is still a major public health concern. Vaccination is one of the most effective strategies for preventing and reducing the prevalence of viral infectious diseases, including HFMD, and many approaches have been tested in order to develop safe and efficient EV71 vaccines [10].

The genome of EV71 encodes a single large polyprotein, which, during infection, is first cleaved into three smaller polyproteins (P1 to P3). The P1 polyprotein is then cleaved by protease 3CD to generate four structural proteins (VP1, VP2, VP3 and VP4) and seven nonstructural proteins. The structural proteins constitute the viral capsid, and the nonstructural proteins participate in virus replication [11–13]. Of the four structure proteins, VP1 is the most important, and several linear immunodominant epitopes have been identified in this protein [10, 14–16].

Virus-like particles (VLPs) are structurally similar to native virions but are non-infectious due to the lack of a virus genome [17–19]. Recently, recombinant VLPs-based strategies have been used for vaccine development. Numerous types of VLPs have been produced by taking advantage of the ability of capsid and envelope proteins of different viruses, including EV71, to self-assemble and form organized structures [20–30]. Commercial VLP-based vaccines have been developed and used successfully to prevent infections with human hepatitis B virus and papillomavirus [31].

The structural polyprotein Pr55gag of the human immunodeficiency virus type 1 (HIV-1) directs the assembly of the virion. The Pr55gag protein is the precursor of four major structural proteins, MA (p17 matrix), CA (p24 capsid), NC (p7 nucleocapsid), and LI (p6 linker), as well as the small spacer peptides p1 and p2 [32]. The unprocessed gag precursor itself is sufficient for the release of non-infectious VLPs in yeast, insect, and mammalian cell expression systems, in the absence of any other viral proteins. In addition to their ability to self-assemble, Pr55gag-based VLPs can also deliver foreign polypeptides and can therefore be used directly as immunogens and as carriers for foreign antigens. Two types of gag-based VLPs have been established [33]: type I VLPs, which are made by integrating or fusing small epitopes with the C-terminus of the gag polyprotein, and type II VLPs, which are made by incorporating foreign proteins at the outer surface of the particle.

In the present study, we constructed and characterized EV71 VLPs that were made based on a type I gag VLP strategy and produced in insect cells. The VLPs were made using full-length gag molecules fused at their C-terminus with EV71 VP1. We first tested whether fusion of the EV71 VP1 gene to the C-terminus of gag results in incorporation as an in-frame fusion. We then found that immunization with these VLPs generate a specific cellular and humoral immune response in mice. Furthermore, we found that injection of female mice with this type of VLP via the intramuscular (i.m.) route provided effective protection of their newborn offspring against challenge with a lethal dose of EV71. Antisera against these VLPs were also able to passively protect newborn mice against challenge with a lethal dose of EV71.

## Material and methods

### Cells and virus

RD (rhabdomyosarcoma) cells were purchased from ATCC and cultured in Dulbecco’s modified Eagle medium (DMEM) supplemented with 10% fetal bovine serum. TN5 cells were cultured in SF900 II insect cell culture medium.

### Preparation of recombinant baculoviruses

The VP1 gene and gag gene fragments were obtained by PCR amplification from the plasmid PCAGGs-VP1 and PCAGGs-gag, respectively (kindly provided by Dr. Ching from Emory University). The gag-VP1 fusion gene was amplified using an overlapping-PCR method. The primers used were as follows: P1, CCGGATTCATGGGAGATAGGGTGGCAGATGTAAT; P2, GATCACCACTCTTGGATCCGGTGGCGGCGGCGTGAGAAAC; P3, GTTTCTCACGCCGCCGCCACCGGATCCAAGAGTGGTGATC; P4, CCCAAGCTTTTACTAACTGGTCTCCTCCAAAGAGAGAAT. The gag and gag-VP1 genes were each cloned into the pFastBacI vector (Invitrogen). The resultant pBac-gag and pBac-gag-VP1 plasmids were used to generate gag and gag-VP1 baculoviruses. The recombinant baculoviruses were obtained and amplified by infecting TN5 cells.

### Purification of the EV71

EV71 was propagated in RD cells. Briefly, virus harvested from infected RD cells was concentrated by precipitation with 8% polyethylene glycol 8000 (Sigma) and then centrifuged at 100,000 × *g* for 4 h through a 15–50% discontinuous sucrose gradient. The layer containing the concentrated virus was pelleted at 100,000 × *g* for 1 h and then resuspended in phosphate-buffered saline (PBS).

### Production and purification of VLPs

TN5 cells were infected with gag-VP1 baculovirus or gag baculovirus at a multiplicity of infection of 5, and the culture medium containing VLPs was collected at 72 h postinfection. Cell debris was removed by high-speed centrifugation, and the supernatant containing the VLPs was concentrated using a GE QSM-03SP/50quixstandTM Benchtop System and further purified by ultracentrifugation at 28,000 rpm for 2.5 h in an SW-41 rotor (Beckman) through a 15-50% discontinuous sucrose gradient. The layer containing the purified VLPs was suspended and washed with PBS, centrifuged at 28,000 rpm for 30 min, and resuspended in PBS. A BCA assay kit was used to determine the protein concentration of the VLP preparation.

### Transmission electron microscopy (TEM)

TEM was performed as described previously [34]. Briefly, VLPs were negatively stained with 1% potassium phosphotungstate and further examined using a Hitachi-H7500 transmission electron microscope (Hitachi, Ltd., Tokyo, Japan).

### VLPs analysis by Coomassie blue staining and Western blot assay

Briefly, VLPs were subjected to 12% sodium dodecyl sulfate–polyacrylamide gel electrophoresis (SDS-PAGE) and followed by Coomassie blue staining and Western blotting. The primary antibody for the Western blot assay was mouse anti-VP1 monoclonal antibody or anti-gag polyclonal antibody. The secondary antibody was horseradish peroxidase (HRP)-conjugated goat anti-mouse IgG. Protein bands were detected using ECL Detection Reagent (Sigma).

### Immunization of mice

Specific-pathogen-free female BALB/c mice were purchased from the animal center of the Fourth Military Medical University (Xi’an, China). Animal experiments were performed according to the current experimental protocols involving animal study approved by the Institutional Animal Care and Use Committee of the Fourth Military Medical University. Six-to-eight-week-old female BALB/c mice were randomly divided into four groups (10 mice in each group), and each mouse was immunized twice at 4-week intervals by intramuscular injection with 10 μg of gag VLPs, 10 μg of gag-VP1 VLPs, 10 μg of inactivated EV71 (in-EV71) or 100 μL of PBS. Blood samples were taken through retro-orbital bleeding two weeks after each immunization (week 2 and week 6) and also collected at the end of weeks 8, 10 and 12. The sera were inactivated at 56 °C for 30 min and stored at -80 °C.

Three or four mice in each group were sacrificed, and their splenocytes were isolated at week 12 to detect cellular immune responses. The remaining mice were used to mate and breed neonatal mice for lethal virus challenge four weeks after the booster immunization, and the neonatal mice were monitored for 15 days.

### Total anti-EV71 and anti-gag IgG response detected by ELISA

EV71 or gag-specific IgG titers in the mouse sera were determined by ELISA. In brief, a 96-well plate was coated overnight at 4°C with purified EV71 or gag protein at a concentration of 0.8 μg/well. After three times washing with 200 μl of PBST, the plate was blocked with 100 μl of 2% BSA for 1 h at 37°C. After another three washes with PBST, serum samples were serially diluted and added to the wells. The plates were incubated at 37 °C for 1 h, after which HRP-conjugated goat anti-mouse IgG was added as a secondary antibody. After three washes, the color was developed using TMB/HCL and the optical density at 450 nm (OD_450_) was recorded.

### EV71 virus-neutralization assay

For measurement the neutralization titer of serum from immunized mice, RD cells were plated in a 96-well cell culture plate and allowed to attach overnight until the cell density reached 80%. Serum was serially diluted twofold (from 8 to 256) with DMEM containing 2% FBS, and the same volume of purified EV71 virus (100 TCID_50_) was then added to the diluted serum and mixed. The serum-virus mixture was incubated at 37 °C for 1 h to neutralize the infectious virus and then added to the cells, which were cultured for another 3 days. The highest dilution that resulted in a 50% reduction of CPE was considered the neutralization titer.

### Cytokine detection by ELISA assays

To detect the cytokines produced after immunization, splenocytes were isolated from each immunized mouse, mixed, and cultured in 96-well microplates (Millipore, Billerica, MA, USA) at a density of 2 × 10^4^ cells/well with 100 μl of RPMI 1640 containing 10% FBS and 10 μg of inactivated EV71 virus or gag-peptide complex (Huaxia Yuanyang Tech, Beijing, China) for stimulation. After incubation at 37°C for 48 h, the plate was centrifuged at 1000 rpm for 5 min, and the cell culture supernatant was collected carefully and assayed for interleukin (IL)-2, IL-4, IL-10, and interferon (IFN)-γ, using cytokine ELISA kits (eBioscience).

### Enzyme-linked immunospot (ELISPOT) assays

The production of IL-4 and IFN-γ was also measured using the ELISPOT method. Briefly, 96-well microplates were coated with anti-IL-4 or anti-IFN-γ monoclonal antibody and incubated in a humidified box overnight at 4°C. Plates were blocked with 2% BSA for 1 h at 37°C, and 5 × 10^5^ splenocytes were then added to each well. Subsequently, inactivated EV71 virus at a concentration of 200 μg/ml was added to stimulate the effector cells for 48 h. After three washes, biotin-labeled anti-IL-4 or anti-IFN-γ antibody was added. After the spots were developed and dried, spot-forming cells were counted using an automated ELISPOT reader (CTL ImmunoSpot analyzer, Cellular Technology Ltd, USA).

### Virus challenge

Four weeks after the second immunization, immunized female mice were allowed to mate. The EV71 challenge strain was diluted to 10^6^ PFU /ml, and each of the neonatal mice was challenged orally with 10 μl of the virus within 3 days after birth. Neonatal mice were monitored daily for 15 days, and the mortality rate was recorded.

### Passive protection assay

A passive protection assay was performed to further evaluate the protective efficacy of serum antibodies raised by vaccination. One-day-old newborn mice (6 mice per group) were inoculated intraperitoneally (i.p.) with sera from mice immunized with gag-VP1 VLPs, inactivated EV71 virus or PBS. Four hours post-inoculation, the suckling mice were challenged i.p. with ten times the 50% lethal dose (10 LD_50_) of EV71. All mice were monitored daily for clinical symptoms and death for 15 days after challenge.

### Statistical analysis

Data were expressed as the average of three independent experiments and analyzed by one-way ANOVA, with *p*-values less than 0.05 considered significant.

## Results

### Incorporation of VP1 into VLPs

**Fig. 1:**
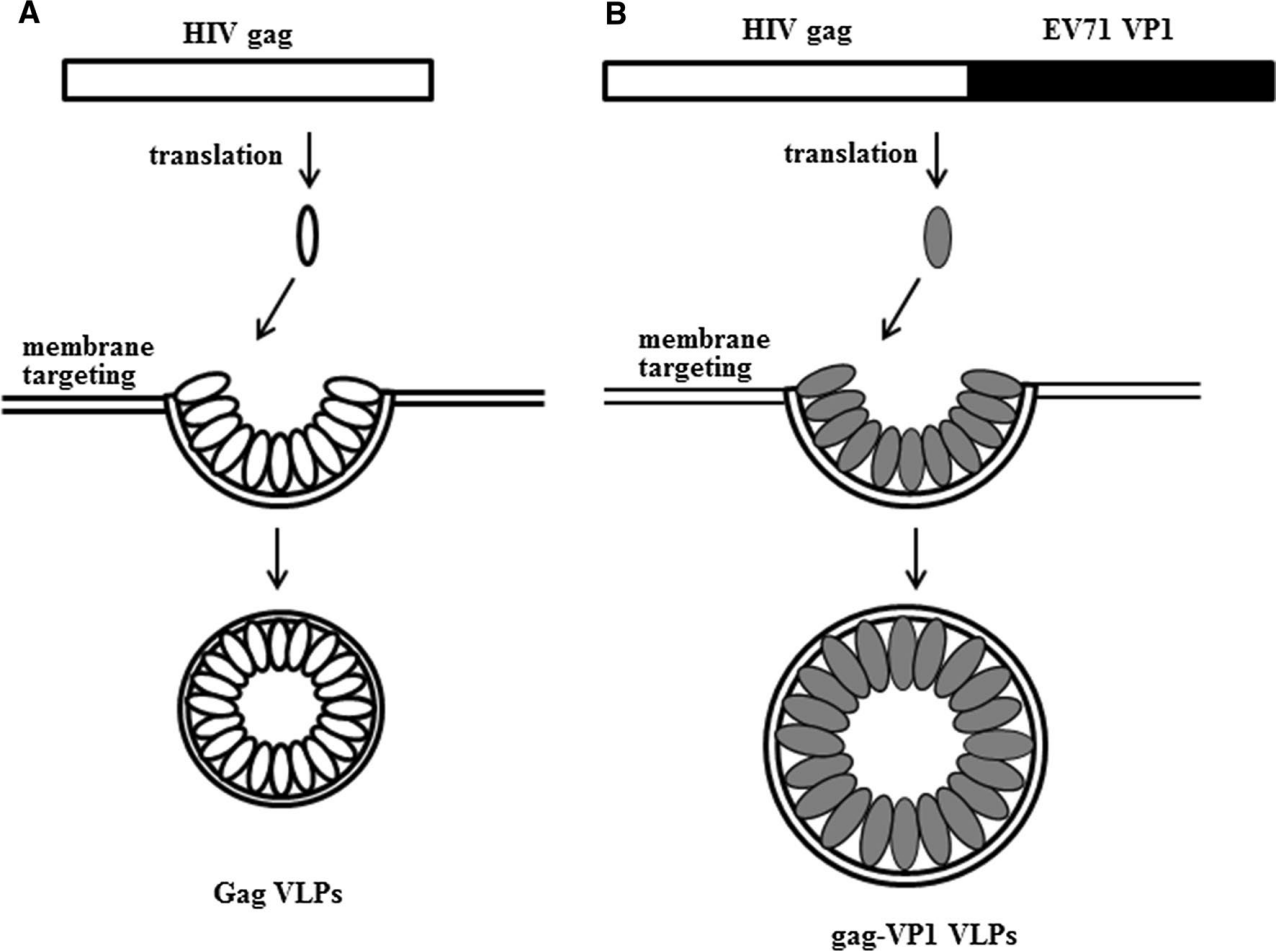
Schematic representation of the gag and gag-VP1 polyprotein and VLPs formation. (A) Cartoon representation of the formation of VLPs (B) Cartoon representation of the formation of gag-VP1 VLPs

**Fig. 2:**
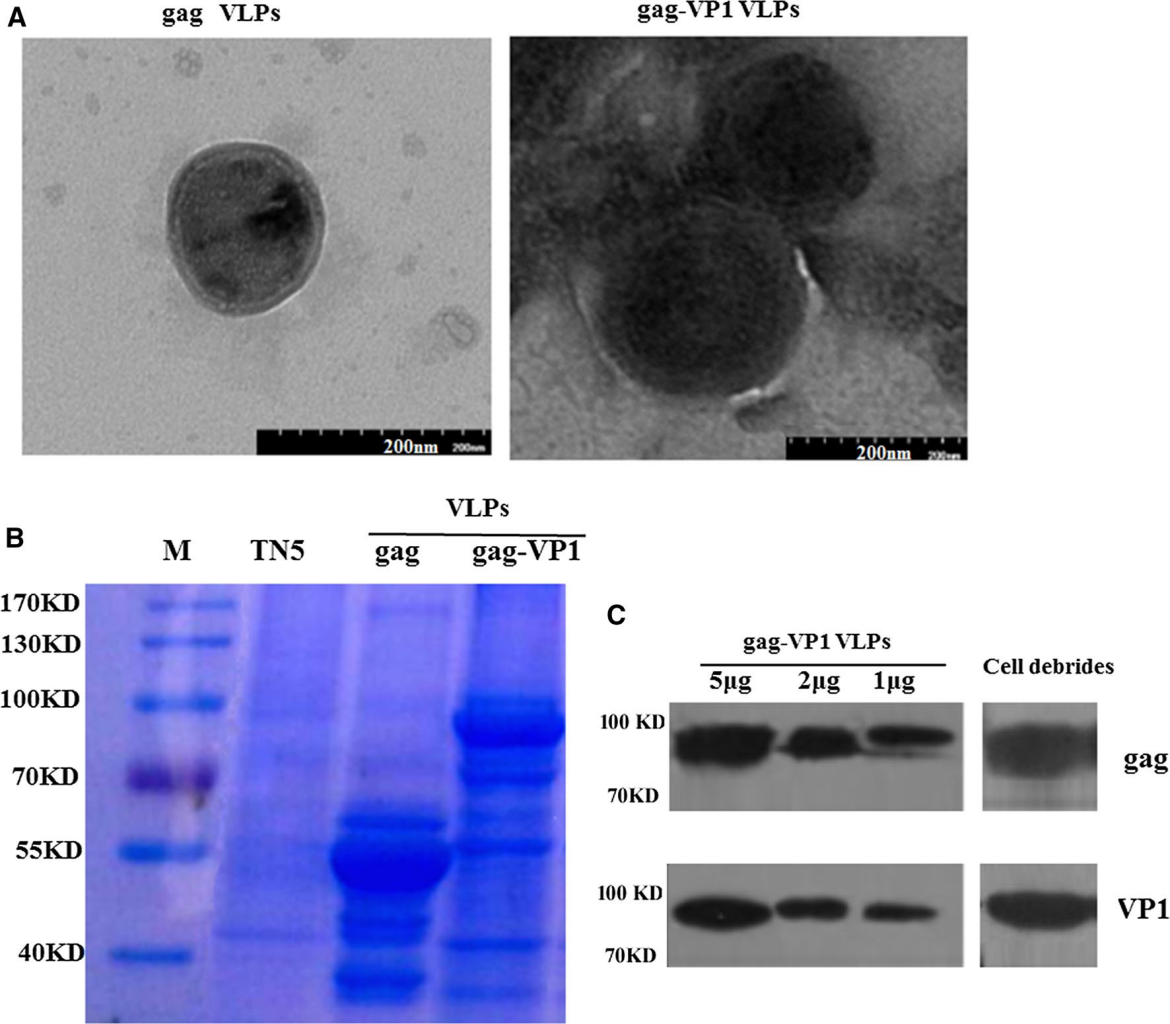
Characterization of gag-VP1 VLPs. (a) Electron microscopy detection of gag-VP1 VLPs. Purified gag VLPs and gag-VP1 VLPs were stained with 1% uranyl acetate, and after washing, VLP samples were observed under a transmission electron microscope. (b) Detection of gag-VP1 VLPs by Coomassie blue staining. The TN5 cell lysate, purified gag VLPs, and purified gag-VP1 VLPs were analyzed by SDS-PAGE and then stained with Coomassie blue. (c) Characterization of gag-VP1 VLPs by Western blot analysis. M, molecular weight markers

DNA sequences encoding heterologous antigens can be fused with gag to generate chimeric gag molecules with a gag-pol frameshift signal that can be used to produce chimeric VLPs. A diagram depicting the formation of control SIV gag VLPs and chimeric gag-VP1 VLPs is shown in Figure 1. The morphology of the VLPs was examined using an electron microscope, and as shown in Figure 2A, gag-VP1 VLPs exhibited a spherical morphology similar to gag VLPs, but the former formed slightly larger VLPs, with a diameter about 150-200 nm. Coomassie blue staining and Western blot analysis were performed to examine the protein profiles of the VLPs. As shown in Figure 2B, the gag-VP1 fusion protein was readily detected in gag-VP1 VLPs by Coomassie blue staining. Chimeric antigens were also detected by immunoblotting using monoclonal antibodies specific for VP1 and HIV gag p24. As shown in Figure 2C, both the VP1 protein and gag were detected in gag-VP1 VLPs. Therefore, the above result indicated that infection with the gag-VP1 recombinant baculovirus (rBv) resulted in the formation and release of VLPs.

### EV71-specific humoral responses in VLPs-immunized mice

**Fig. 3:**
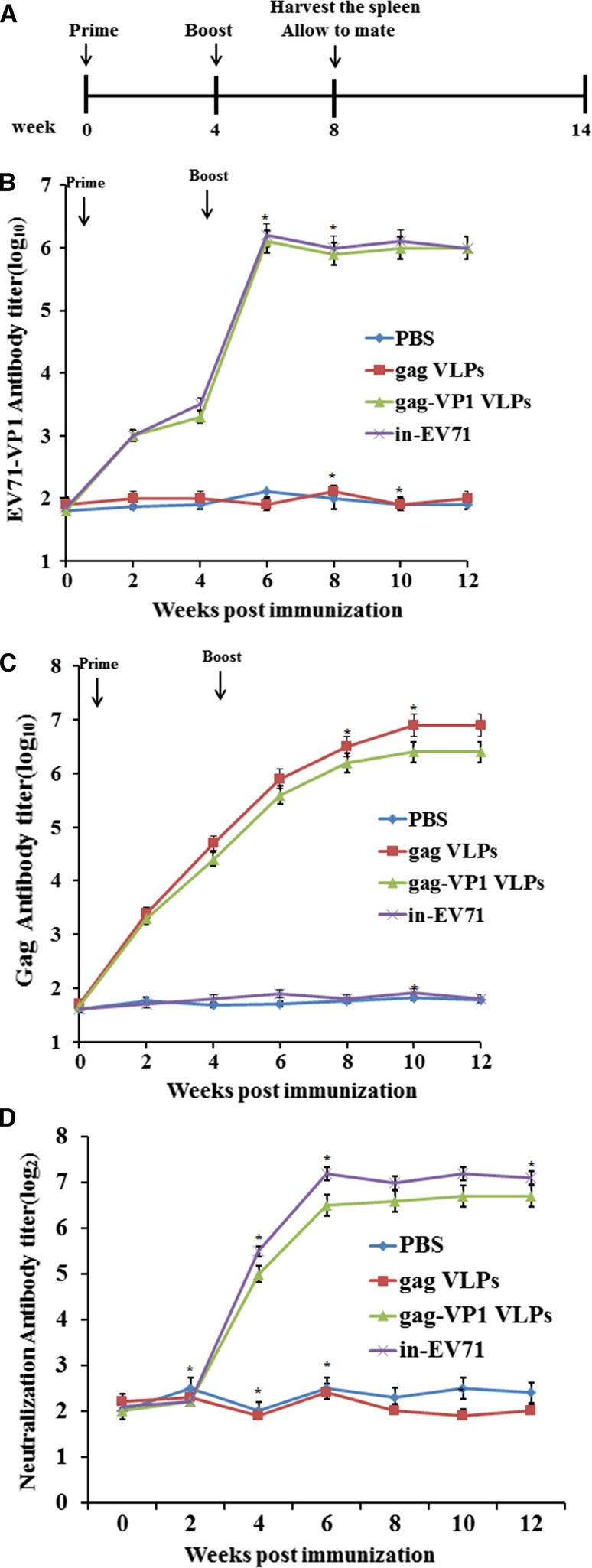
Induction of a humoral immune response by immunization with gag-VP1 VLPs. BALB/c mice were divided into groups of 10 mice each and then inoculated by the i.m. route with 100 μl of PBS, 10 μg of gag VLPs, 10 μg of gag-VP1 VLPs, or 10 μg of in-EV71. Mice in all groups received a booster immunization at the end of week 4. (A) Immunization schedule. (B) Sera were collected 2 weeks after each immunization and used to detect the production of EV71-specific IgG antibody by ELISA. (C) Sera were collected 2 weeks after each immunization and used to detect the production of gag-specific IgG antibody by ELISA. (D) NAb titers were determined using a neutralization assay

An ELISA was performed to investigate anti-EV71 antibody responses induced by gag-VP1 VLPs. Female BALB/c mice were immunized i.m. with purified gag-VP1 VLPs, gag VLPs, or inactivated EV71 (in-EV71) (10 μg/mouse) and received a booster immunization 4 weeks later. Mice immunized with PBS were used as negative controls. For serological analysis, blood was collected at the end of weeks 0, 2, 6, 10 and 12. The results of ELISA analysis (Fig. 3B and C) showed that gag-specific antibody was detectable 2 weeks after the primary immunization with gag VLPs or gag-VP1 but antibodies specific for EV71 were only detectable in mice immunized with gag-VP1 VLPs and in-EV71. The antibody titers increased after the booster immunization and were detectable until week 12. The EV71 VLPs and in-EV71 elicited similar levels of IgG antibodies. In contrast, mice inoculated with PBS showed almost no anti-VP1 or anti-gag response.

A neutralization assay was also performed to detect neutralizing antibodies (NAbs) induced by the gag-VP1 VLPs. Two weeks after the primary immunization, serum from mice inoculated with gag-VP1 VLPs showed an NAb titer of 1:32, and 2 weeks after the booster immunization, the NAb titer reached almost 1:128, which is slightly lower than the in-EV71-induced NAb titer (Fig. 3D). In contrast, serum from mice inoculated with PBS or gag VLPs displayed almost no anti-EV71 NAbs. These results clearly demonstrate that gag-VP1 VLPs can induce a strong humoral immune response in mice.

### Cytokine secretion by EV71-specific T cells

**Fig. 4:**
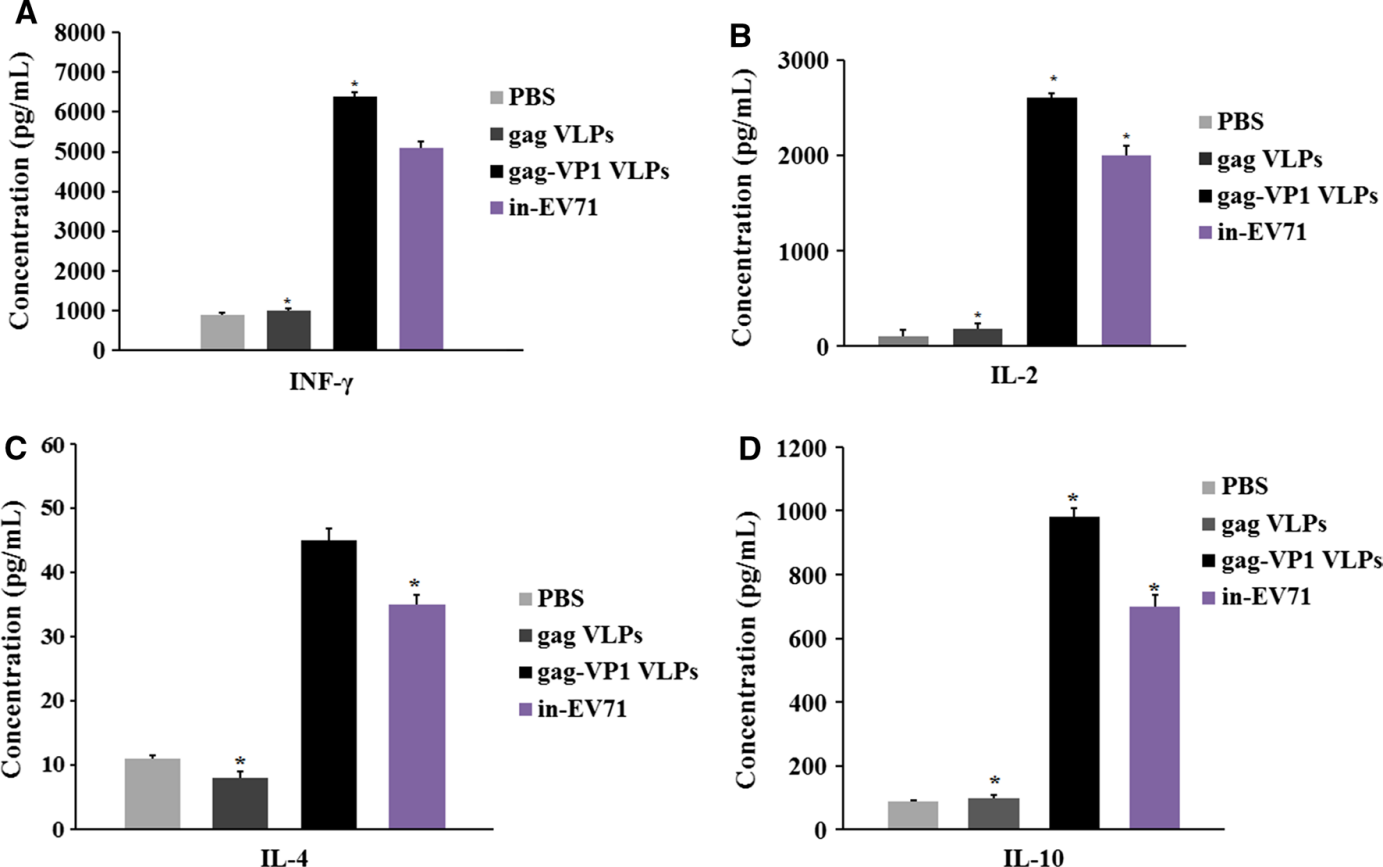
EV71-specific cellular immune responses induced by immunization with gag-VP1 VLPs or in-EV71. Splenocytes were isolated and stimulated with EV71 4 weeks after the second immunization. The cell culture supernatant was collected 48 h later, and a cytokine ELISA was performed to measure the secretion of (A) IFN-γ, (B) IL-2, (C) IL-4, and (D) IL-10. The data represent the average for five mice in each group

**Fig. 5:**
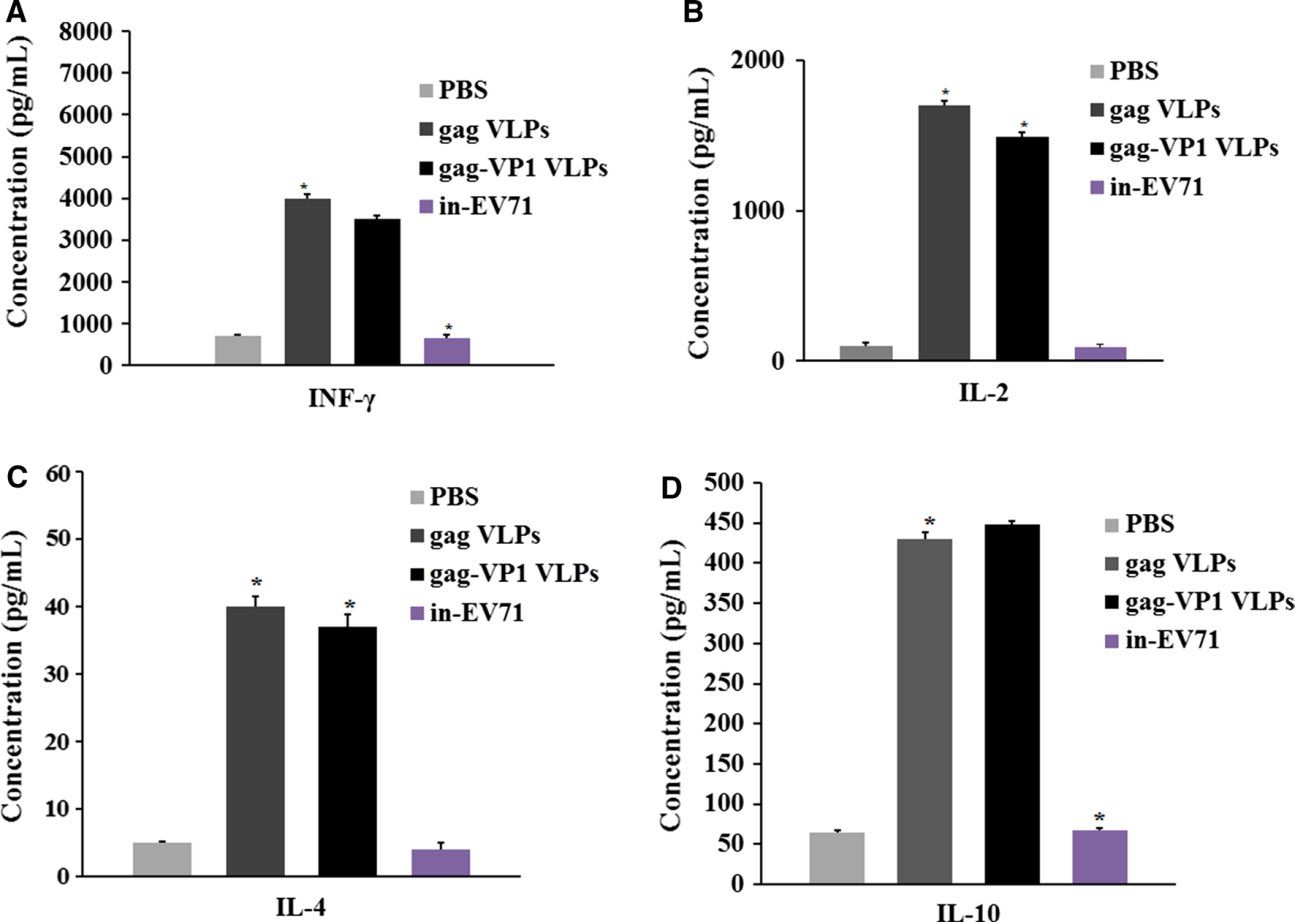
Detection of gag-specific cellular immune responses in gag-VP1- and gag- immunized mice. Splenocytes were isolated and stimulated with gag-peptide complex 4 weeks after the second immunization. The cell culture supernatant was collected 48 h later, and cytokine ELISA was performed to measure the secretion of (A) IFN-γ, (B) IL-2, (C) IL-4, and (D) IL-10. The data represent the average for five mice in each group

To examine the T cell response induced by gag-VP1 VLPs or gag VLPs, we measured the cytokines produced by VLP-stimulated spleen cells using a cytokine ELISA. Figure 4 shows that, after EV71 stimulation, EV71-specific cytokines were only detectable in mice inoculated with gag-VP1 VLPs or in-EV71, and the levels of IFN-γ, IL-2, IL-4, and IL-10 were much higher in the group immunized with gag-VP1 VLPs than in the group immunized in-EV71. In contrast, the level of gag-specific cytokine secretion was similar between the gag VLPs group and the gag-VP1 group (Figs. 4, 5).

### T cell ELISPOT assay

**Fig. 6:**
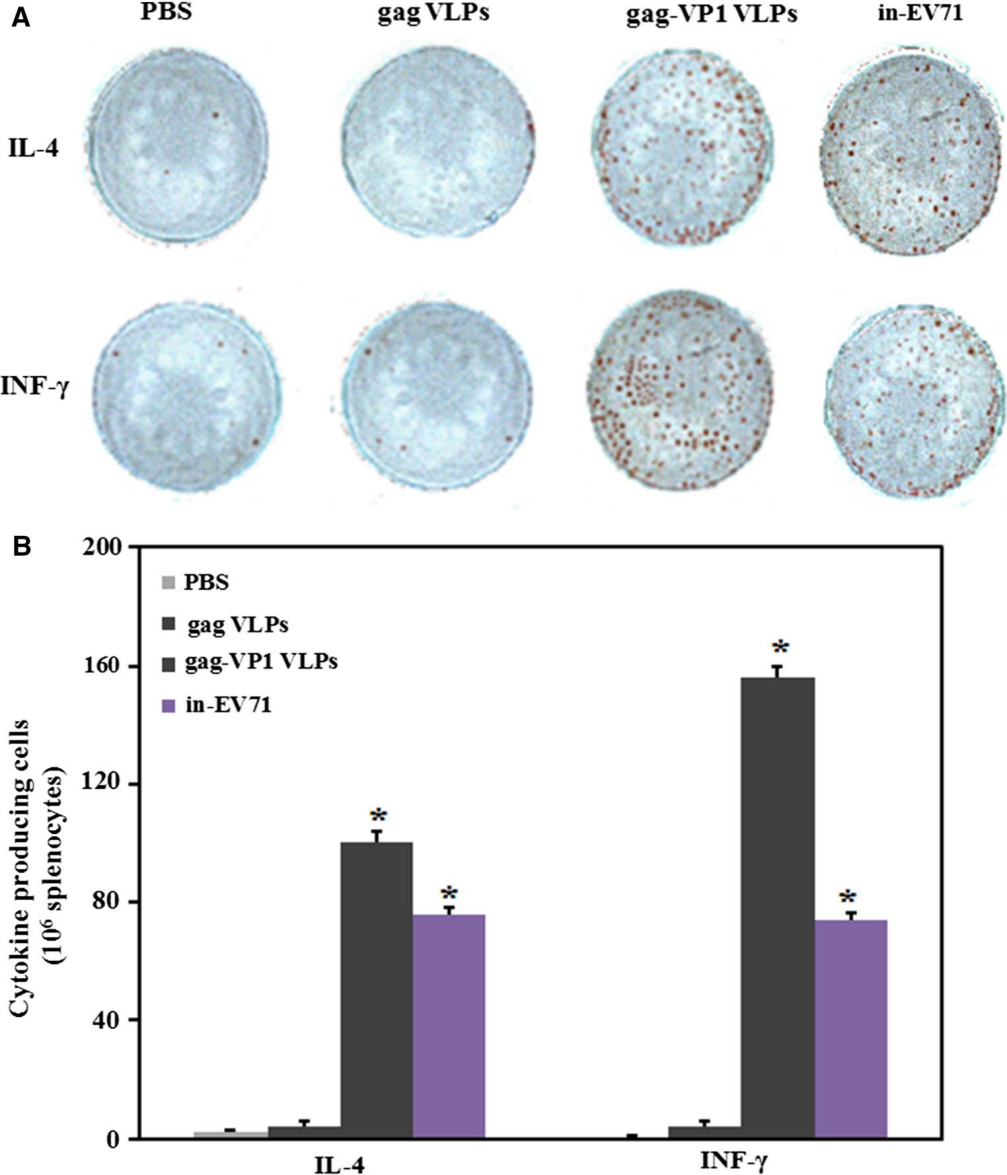
EV71 VP1-specific T cell responses detected using an ELISPOT assay. (A) Spots on the ELISPOT plate showing IFN-γ- and IL-4-secreting T cells in mouse spleens. (B) The number of EV71-specific T cells in mouse spleens after treated with different immunization regimens. Results for triplicate wells are shown

To further evaluate EV71-specific T cell responses induced by different vaccination regimens, spleen cells from immunized mice were harvested, and IFN-γ and IL-4-secreting cells were detected by ELISPOT assay after stimulation with EV71 VLPs. As illustrated in Fig. 6, mice in the gag-VP1-immunized group produced significantly more IFN-γ- and IL-4-secreting T cells than those in the in-EV71-immunized group, confirming that gag-VP1 immunization generated stronger specific T cell responses than in-EV71 immunization.

### Protection of newborn mice against challenge with lethal EV71 virus

**Fig. 7:**
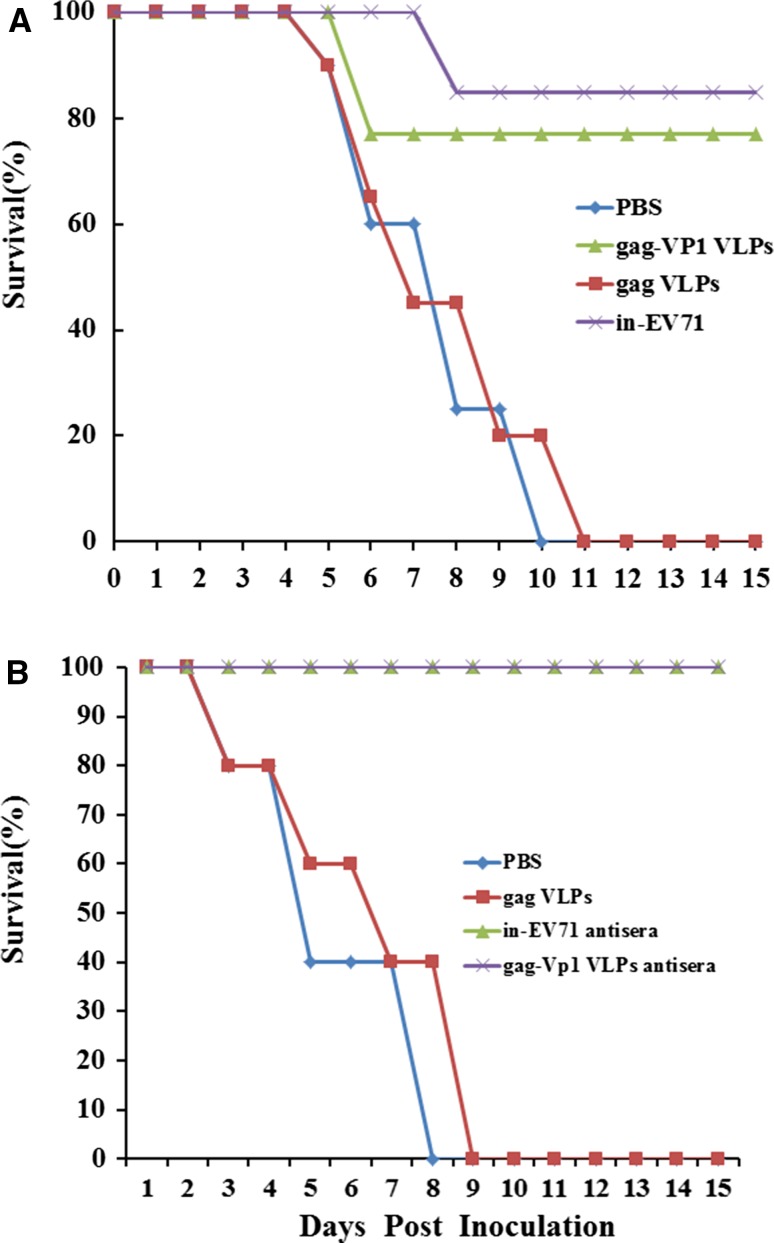
Passive protection of newborn mice against challenge with a lethal dose of EV71. (A) Newborn mice born to immunized female mice were infected by the intraperitoneal route with 10^6^ PFU of the homologous EV71 strain, and the mice were monitored daily for 15 days and the survival rate was recorded. (B) One-day-old BALB/c mice (6 mice per group) were inoculated intraperitoneally with anti-VLPs serum or PBS. Within 4 h of inoculation, each mouse was challenged intraperitoneally with 10 LD_50_ of the homologous EV71 virus. Mortality was monitored and recorded daily for 15 days

To investigate the passive protection efficacy against homologous virus challenge, adult female mice were injected intramuscularly with gag-VP1 VLPs, in-EV71, gag VLPs or PBS, and allowed to mate after the second immunization. The neonatal mice were then challenged within 72 hours of birth with a lethal dose of EV71 and subsequently monitored daily for survival and clinical symptoms for 15 days. Newborn mice born to female mice that had been pre-immunized with PBS or gag VLPs gradually developed severe manifestations including reduced mobility, a ruffled coat, and limb weakness, and eventually died, with a final mortality rate of nearly 70% at the end of day 15. In contrast, all of the neonatal mice born to mice injected with gag-VP1 VLPs survived, with only transient or minor symptoms. The immune-protective efficacy reached nearly 80%, which is slightly lower than the survival rate of the group immunized with inactivated EV71 (Fig. 7A).

In another experiment, immune sera from animals that had been given gag-VP1 VLPs or inactivated EV71 were injected i.p. into one-day-old neonatal mice, which were then challenged with 10 LD_50_ of EV71 within 4 h. As shown in Fig. 7B, control mice injected with sera from mice inoculated with PBS or gag VLPs started to show disease symptoms at day 4, and all died by day 8. In contrast, mice inoculated with immune sera from mice inoculated with gag-VP1 VLPs or inactivated EV71 remained healthy and survived until the end of day 15. These results demonstrate that antibodies induced by gag-VP1 VLPs or inactivated EV71 could protect newborn mice against EV71 infection.

All of the above results show that maternal neutralizing antibodies were able to protect newborn mice against lethal challenge by EV71.

## Discussion

HFMD has been ranked as one of the infectious diseases with the highest incidence in China, and was included in class C of infectious diseases in 2008. During the years 2008-2012, 7,200,092 probable cases of HFMD were reported according to the China CDC surveillance system [35]. EV71 and coxsackievirus A16 (CA16) are the leading causes of HFMD outbreaks in China; however, clinical symptoms induced by EV71 are much more severe than those induced by CA16. Most immune strategies against EV71 target its structure protein VP1, and the focus of vaccine development in this field has been based mainly on inactivated viruses and VLPs. Although the EV71 inactivated vaccine successfully entered the market in China in 2015, the production of inactivated EV71 vaccine was at a relatively low level [36], and there was concern about its safety profile. As an alternative EV71 vaccine platform, VLPs are safer than inactivated vaccines because they do not contain the viral genome.

HIV-1 Pr55gag-based VLPs are prime candidates for delivering foreign polypeptides, and there are two ways to produce VLPs using HIV gag. In the present study, we constructed an in-frame gag-VP1 fusion to generate rBv gag-VP1 using the Bac-to-Bac system, and this recombinant virus was used to infect insect cells to form type I VLPs based on the ability of the gag protein to self-assemble.

The gag-VP1 VLPs were then used to immunize BALB/c mice, and serum samples from immunized mice were collected and assayed. The titer of EV71-specific IgG clearly increased 2 weeks after booster immunization, as measured by ELISA, and antibodies remained detectable for more than 12 weeks. More importantly, EV71-specific neutralizing antibodies induced by gag-VP1 VLPs were able to protect mice against a lethal challenge with EV71. These results demonstrated that immunization with gag-VP1 VLPs induces a high-level and persistent antibody response. Humoral immune responses are essential for protecting against EV71 infection; however, several studies have also indicated that the cellular immune response affects the severity of HFMD [37, 38]. Different cytokines are involved in the activation of two distinct major subsets of T cells. In the present study, we isolated splenocytes from mice immunized with VLPs and detected INF-γ and IL-2, which are released by Th1 cells, and IL-4 and IL-10, which are released by Th2 cells, showing that both Th1-type and Th2-type responses were induced. VLPs immunization induced a higher cytokine response than immunization with the inactivated EV71 vaccine, indicating that gag-VP1 VLPs immunization can stimulate the production of cytokines associated with both the Th1 and Th2 immune responses. The results of the cytokine ELISA were confirmed by ELISPOT assay.

We also observed *in vivo* protection by gag-VP1 VLPs against viral challenge in animal models. Newborn mice born to mothers that had been pre-immunized with gag-VP1 VLPs or pre-inoculated with gag-VP1 antisera were able to survive until the end of day 15 post-challenge.

In summary, gag-VP1 VLPs was successfully produced using an insect cell expression system. The induction of both humoral and cellular responses by this vaccine was evaluated, and its ability to confer passive protection to newborn mice against a lethal dose of EV71 was determined. The VLPs elicited a strong antibody response as well as a cellular immune response resulting, in antisera with high neutralization titers. The antisera were able to protect newborn mice in a passive protection experiment. As VLPs are considered safer than inactivated virus vaccines, the use of gag-based VLPs containing EV71 VP1 should be considered for HFMD vaccine research.
